# Professional Services and Professional Fees

**Published:** 1884-12

**Authors:** Edwin T. Darby


### Independent Practitioner.

VOL. V. DECEMBER, 1884. NO. 12.

### Original Communications.

### PROFESSIONAL SERVICES AND PROFESSIONAL FEES.

BY EDWIN T. DARBY, M. D., D. D. S.

READ BEFORE THE CENTRAL DENTAL ASSOCIATION OF NORTHERN NEW JERSEY.

*Mr. President and Gentlemen:*

I appear before you this evening to fulfill a promise made the worthy chairman of your executive committee some months ago. But in doing so I have been beset by one of the greatest difficulties which it is the lot of an essayist to encounter, namely; the selection of a subject. I have spent more anxious thought in the effort to decide upon a theme appropriate to this occasion than would have prepared a better paper than the one to which I now invite your attention.

The subject which I have chosen is peculiar, in that it has seldom been discussed by our societies, but it cannot fail to be of some little interest, because it relates to the chief object of our toil. As a sentiment it sounds well to say that we work for the good of the public. As a fact, we work for the good of ourselves, and those dependent upon us.

Professional services, like articles of merchandise, have a value which is usually relative, but is, nevertheless, determined by the law of demand and supply. Most articles which are bought and sold are subject to this law. Scarcity of anything merchantable enhances its value. Diamonds are valuable because they are rare. If it were possible to crystallize the coal beds of Lamokin, and Scranton, and New Castle, diamonds would sell for six dollars per ton instead of two hundred per carat.

Fine works of art are valuable because they are rare. If the paintings of Michael Angelo, and Raphael, and Murillo, and West, and Turner were as common as the cartoons of *Puck*, they would be as valueless.

Fine tapestries, and laces, and cloths, and bronzes, and watches, and mechanical appliances are valuable because they are the product of skilled labor, and skilled labor is rare and commands high wages; while the former may be absolute the latter is usually relative. The value of all articles of merchandise is determined by the law of supply and demand. It is the quantity of an article produced, and the demand for such an article, which make it valuable or otherwise. The same law will in a measure apply to professional services.

Professional services, like articles of merchandise, have a value which is either absolute or relative, and is determined by the quality, demand, and supply.

The same law applies with equal force to all commodities. When there is a dearth in the wheat or corn crop, the price is high. When there is an excess over the demand needed for consumption, the price is correspondingly low. The demand and supply regulates the price, and the price is usually the value. Ordinarily speaking, a thing is worth all that it will bring, but there are fancy or fictitious values in contradistinction to real or intrinsic. It is hardly reasonable to suppose that a horse is worth $40,000, or a dog $5,000, or a cow $2,500; but cows, and dogs, and horses have been sold at these prices, and the inference is that they are worth it.

Society demands some standard of valuation, and when applied to the commodities of life it is regulated by the law above referred to.

The services of professional men do not, strictly speaking, come under this head, for every man is, in a certain sense, a law unto himself. It is true that some professions have their scale of prices, or fee bills, as they are termed. The lawyer has a certain fee for drafting wills, and deeds, and mortgages, and replevins, and judgments, but these are formulated writings which can be deputized to another; hence a fixed price is usually charged for such writings. When the question is one of opinion, the fee is variable, and may be high or low, just in proportion as his services are much or little sought after. Strictly speaking, a lawyer's fee is what he charges for his individual services; the value of his opinion, the price of his retention in a case. His retaining fee may be $5 or $50,000, and is determined by the importance of the case, the labor required, and in some instances by the fatness of his client's pocket-book. Physicians have a fee-bill, or scale of prices, but they seldom adhere to it, and with most practitioners it is a dead letter. It stipulates what shall be the minimum charge for an office or residence visit, but does not say what the maximum charge shall be. The public have just about as correct an idea of its meaning as they have of a gas meter after reading the directions on a gas bill.

As a rule, physicians are underpaid. No class of professional men do more work gratuitously than does the conscientious practitioner of medicine. He is at the beck and call of the poor and lowly, and in the earlier days of his professional career receives more "God bless yous" than substantial fees. It is only when he becomes celebrated that he begins to reap what he has sown during the years of his obscurity. One of the most celebrated physicians of Philadelphia once told me that when he began the practice of his profession in that city his fee for a visit was twenty-five cents, and he was thankful if he collected that.

The men whose names are historic in medicine, and those who are now celebrated, have risen from obscurity, and have received small fees in the beginning. To the beginner a small fee is better than no fee at all; a half loaf better than none; and the practitioner who has worthy aims is better content to attend the poor for insignificant fees than to sit in his office and wait for the calls of the rich. The medical man who did most for small fees at the beginning is generally he who has done most for large fees in after life. A century ago a young man of obscurity graduated from the University of Edinburgh, and at once settled in the city of London. He paid six shillings and six pence for his room rent, and received from his practice the first year five pounds (£5); but in after years when swaying the surgical scepter of England, as Sir Astley Cooper, his practice in a single year amounted to £23,000.

If statistics are reliable the American public receive their medical attention at a very small cost *per capita.* Recent calculations have shown that in the city of Philadelphia, where there are, perhaps, a greater number of physicians in proportion to the population than in any city in America, the average annual income of physicians from their practice is less than $1,200.

The quality of professional service determines its value. It sometimes happens that the public obtain the services of skillful men for less than they are worth, but, on the other hand, they often pay exorbitant fees for ignorance or malpractice. There is, perhaps, nothing for which money is paid that is so uncertain in its value as professional service, nor is there any subject upon which the public show a greater amount of ignorance than in the matter of professional skill. No better illustration of faith could be found than is daily witnessed by the medical man. The public opens its great mouth to skillful and unskillful alike, and allows the quack and charlatan to pour, as Voltaire has said, "drugs of which they know little into stomachs of which they know less." Society, like a wagon wheel, runs in ruts. It not infrequently happens that men of mediocre attainments have become celebrated and affluent, because the tide set in their direction. People patronize a man because the *elite* of the village or city do so, and often pay exorbitant fees for services which are of themselves valueless. It is sometimes more profitable to become fashionable than to become skillful. Who of us cannot recall men whose offices are thronged with admiring patients, joyously paying large fees for inferior services, while his professional neighbor, conscious of superior skill, languishes in poverty and obscurity.

Few people are able to judge of the value of anything outside of their own specialty. Nine-tenths of those who purchase judge of the value by the price asked. They reason that a thing must be good if it be high in price. They apply the same rule to professional services that they would in the selection of an India shawl. It is not the ignorant and superstitious alone who thus estimate values. Some years ago an intelligent physician called upon me to ask the quality of some operations performed for him by a dentist in a little village where he was spending his summer vacation. He employed him because he had leisure, and *presumed* he was skillful. He had no reason to doubt the quality of the service rendered until he paid the bill. The price charged was one dollar per cavity for gold fillings, many of them large. The operations were beautiful; had he paid five or ten dollars each instead of one, he would have been sure the work was good. I recall another case, the reverse of this, but which illustrates the argument. A gentleman about going abroad, to be absent a number of years, asked me to whom he should apply in case he needed services while there. I gave him the names of several whom I believed good men and true. When he returned, some years after, he told me that he had been in the hands of one of the gentlemen, and had five gold fillings inserted, for which he paid the modest sum of $400. He did not question the quality of the work, but thought it just a little dear in price. It is one of the characteristics of humanity that it appreciates most that which costs most, whether it be of money, of labor, or of sacrifice. It has often been said that the professional man has ample opportunities for deception and fraud, and the saying is undoubtedly true. He has it in his power to palm off ignorance for knowledge, poor work for honest service, and may extort from his patient extravagant fees, while another would be satisfied with reasonable ones.

There is, perhaps, no calling in life where innate honesty is more essential than in the practice of dentistry. The dentist can conceal his mistakes and blunders almost as well as the physician; if he be shrewd as well as dishonest, he can deceive his confiding patients at every turn, and it may be months or years before they are aware of it.

During the last quarter of a century great changes have been made in the methods and value of service in our specialty. The introduction of cheap bases for artificial teeth, and the increase of more than eight thousand practitioners of dentistry, have had a tendency to lower the standard of excellence, and to materially affect the price of dental operations. I am not prepared to say that the introduction of rubber, celluloid, and other cheap bases has been a curse to the public, but I am strongly of the opinion that thousands of valuable natural teeth are annually sacrificed, and their place supplied by miserable plates at miserable prices. So great has become the competition in the country, and even in some of our city offices, that whole dentures are furnished at the small sum of ten dollars.

I met a gentleman, a few weeks ago, in the interior of New York State, whom I had known twenty years ago as a reputable practitioner. He said that so great had become the competition in his own vicinity that he was now making whole upper and lower sets of teeth for ten dollars, and others were doing it for less. Gold fillings were inserted for one dollar, and amalgam and other plastics for fifty cents. The demand was for cheap work, and there were more than enough dentists to supply the demand at those low rates. So little skill is required in the construction of these cheap bases that in the past the blacksmith has forsaken his anvil, and the joiner his plane, and with forceps, impression cups, and vulcanizer, he has itinerated the country, supplying the demands of the people.

In mercantile pursuits competition is said to be the life of trade, but its twin sister, over-production, has been the death of many. When the supply exceeds the demand, prices are low, and often ruinously so. Our country is at the present time experiencing the baneful results of over-production. Factories and mills are being closed, and coal mines are being flooded, and the laborer and operative are suffering for employment. History has shown that whenever there has been depression or a panic in business, the professions have had a large influx. Our medical and dental schools have opened the present year with large classes, and will continue to do so until the depression ends.

Of late there seems to be a growing belief that the dental profession offers one of the most lucrative fields in which to labor, and it is sometimes amusing to know the estimate which people place upon our work and our pay. A business man, who had several sons approaching manhood, called upon me recently to ask my advice about one of them whom he thought of educating in dentistry. He said his son leaned toward dentistry, and as it seemed to be an easy life, with big pay, he himself believed that he could not do better than to start him in the "business." My reply to him was to the effect that if he expected his son to have an easy life with a fat purse, he had selected the wrong calling. The two conditions are incompatible. The men who have been successful in dentistry have had laborious lives, sacrificing health, recreation and enjoyment, and, as a rule, dying an untimely death.

The average dentist is poor; poverty sat by his cradle, was his playmate and companion through life, and often follows him to his grave.

If we have fine homes and the comforts which others enjoy, it is because we are diligent in business, prudent in expenditures, and conscientious in our dealings with those who employ us. Notwithstanding we have trials and perplexities (and I sometimes think the dentist has more than others), it is encouraging to believe that the more intelligent of every community appreciate the laboriousness of our lives, and pay our fees cheerfully.

But there comes a period in the life of every dentist who has been successful in attracting a large clientele, when the matter of fees or charges for his services becomes one of the problems which he must solve, In the earlier years of his professional career, when patients are few and his reputation yet unmade, he is better content to accept small fees than to sit in idleness. His modesty in the matter of attainments, and his timidity lest he drive some away in consequence of his charges, prompt him to keep his fees below those of other men engaged in the same calling, and often below their actual value. But when in after years his services are sought by greater numbers, and his appointment book is filled for weeks or months ahead, he begins to feel that his experience has enhanced the value of his service, and he instinctively puts a higher moneyed estimate upon his skill. The tyro may perform a given operation as well as the man who has had twenty years of experience, but the beginner lacks the judgment which twenty years of experience will furnish him. Hence he lacks that which will enable him to decide when and how to perform a given operation. If your services and mine are worth more than the services of the newly-made graduate of one of our colleges, it is because we have had the experience of ten, twenty, or thirty years, and with it the accumulated skill which these years of experience must bring us. How, then, is the man of experience to regulate his charges? We have seen that competition in the country has lessened the standard of excellence, has reduced the price of professional service to that of mechanic's wages, has killed ambition, and has been the cause of the sacrifice of thousands of valuable teeth.

Professional men ought never to compete in anything save excellence.

The fee system of Europe has some features which commend it to our American practice, but it has defects which it is to be hoped will prevent its adoption in this country.

The English fee is a guinea for consultation, extraction, and ordinary stoppings (fillings). For the minor operations it would seem to us an excessive charge, but the expectation is that the average will be made good in the more prolonged or difficult operations. Having a given fee for each sitting, whether it be long or short, the tendency is to make it as short as possible. It is not an unusual thing for a dentist in full practice in England to see from twenty-five to fifty patients in a day, receiving from each a guinea. An American dentist would not feel that he could do justice to half that number. Patients of mine, who have sojourned in England and been in the hands of English practitioners, complain at the aggregate cost of this system of charging. Assuming that English operative dentistry is equal to ours (which it is not), the cost to the patient would be greater than the charge of the average American dentist of ability for a similar amount of work.

The French and German-American dentists have a similar fee. The usual fee in France is a napoleon, or about four dollars American money. The German dentist proper has a mixed way of charging, but the German-American dentist has a minimum charge of fifteen marks, or about three dollars and seventy-five cents of our money, and often doubles it for a sitting of any considerable length.

It is to be presumed that in countries where amalgam and the plastics are more commonly used by the better dentists, this system would work better than with us, who use a larger percentage of gold for filling teeth. Perhaps the most just way of fixing one's charges is by the hour, or the time system, and the testimony of those who have tried it for years is to the effect that it is more satisfactory to the majority of patients. In cities, and among practitioners who confine themselves exclusively, or nearly so, to operations upon the natural teeth, it has much to commend it. It insures to the dentist pay for his time, and time is his stock in trade. It insures to the patient painstaking work, because the operator has no selfish motive to hurry. It prevents misunderstanding in the matter of accounts, for the patient can keep his own reckoning. It is more professional, for it is a charge for time and service, and not for material. It inculcates the adage that time is money, and so prevents loitering and needless conversation. But you tell me that the dilemma is unchanged, and how is the dentist to estimate the value of his services per hour? One man is slow in his movements and gentle in his touch; another is as quick as lightning and accomplishes much more in a given time; hence his services are cheaper to the patient if the price per hour be the same. I am free to admit that there is force in the objection, but the price per hour need not be the same. Every man has a pretty correct idea of the value of his time. He knows, or should know, what income he should receive for a year's service. Let us occupy a moment in details. Of the three hundred and sixty-five days in a year, fifty-two are, by custom, set apart as days of rest, leaving three hundred and twelve days, exclusive of holidays. But no dentists should, and few can, pursue their calling without periods of recreation. A month is too little, but it is better than nothing. Let us subtract, then, forty days for pleasure, leaving two hundred and seventy-two working days. The average dentist, in full practice, stands at his chair seven hours per day (if he does more he dies earlier), making a total of nineteen hundred and four hours. From this a liberal reduction must be made for unavoidable delays and unaccomplished purposes, reducing the number of paying hours in a year to about eighteen hundred and fifty, which at five dollars per hour would amount to $9,250; or at ten dollars per hour to $18,500. These fees may seem high to some, or low and reasonable to others. They are about the average prices charged by dentists, whether it be by operation or by time, and are as low as the public can expect from professional men who devote their lives to the task of saving teeth. Few men engaged in our calling have amassed a fortune, or even a competence; but if the facts were known, it would be seen that the men who have been uniform in their charges and methodical in calculating the value of each moment and hour are those who have accumulated most and served the public best.

---

### LEGAL DECISIONS.

A dentist in San Francisco, who forcibly removed some fillings the payment for which was in dispute, was condemned by the courts to pay damages in the sum of $217.50. It has also been decided that a dentist cannot retain an unpaid-for set of teeth that may come into his hands again for alterations or repairs.

